# Integrin β6 serves as an immunohistochemical marker for lymph node metastasis and promotes cell invasiveness in cholangiocarcinoma

**DOI:** 10.1038/srep30081

**Published:** 2016-07-21

**Authors:** Zequn Li, Siddhartha Biswas, Benjia Liang, Xueqing Zou, Liqun Shan, Yang Li, Ruliang Fang, Jun Niu

**Affiliations:** 1 Department of General Surgery, Qilu Hospital of Shandong University, Jinan 250012, Shandong, China; 2 Key Laboratory of Cardiovascular Remodeling and Function Research, Chinese Ministry of Education and Public Health, Jinan 250012, Shandong, China

## Abstract

Cholangiocarcinoma is a devastating malignancy that is notoriously difficult to diagnose and is associated with a high mortality. Despite extensive efforts to improve the diagnosis and treatment of this neoplasm, limited progress has been made. Integrin β6 is a subtype of integrin that is expressed exclusively on the surfaces of epithelial cells and is associated with a variety of tumors. In the present study, we investigated the expression and roles of integrin β6 in cholangiocarcinoma. β6 upregulation in cholangiocarcinoma was correlated with lymph node metastasis and distant metastasis. Moreover, integrin β6 was identified as a biomarker for the diagnosis of cholangiocarcinoma and an indicator of lymph node metastasis. Integrin β6 significantly promoted the proliferation, migration and invasion of cholangiocarcinoma cells. Furthermore, integrin β6 increased Rac1-GTPase, resulting in the upregulation of metalloproteinase-9 (MMP9) and F-actin polymerization. Taken together, our results indicate that integrin β6 promotes tumor invasiveness in a Rac1-dependent manner and is a potential biomarker for tumor metastasis. Integrin β6 may help to improve the diagnostic accuracy, and targeting β6 may be a novel strategy for the treatment of cholangiocarcinoma.

Cholangiocarcinoma is neoplasm originating from the ductular epithelium of the biliary tree, either within the liver (intrahepatic cholangiocarcinoma) or more commonly from the extrahepatic bile ducts (extrahepatic cholangiocarcinoma). It was first reported by Durand Fardel in 1840, and the incidence of this rare disease is increasing^1^,^2^,^3^,^4^,^5^. The major risk factors for cholangiocarcinoma are age, primary sclerosing cholangitis (PSC), chronic choledocholithiasis, hepatolithiasis, bile duct adenoma, and parasitic biliary infestation^6^. This disease is notoriously difficult to diagnose, and surgical resection is the current therapy of choice. The prognosis of cholangiocarcinoma is poor due to its late clinical presentation and the lack of effective non-surgical therapeutic modalities, with a 5-year overall survival rate of less than 5%, which has not significantly changed in the past 30 years^7^,^8^. Therefore, a better understanding of the molecular mechanisms that are associated with the development of cholangiocarcinoma is needed for the treatment of this devastating disease.

Integrins are major cell adhesion receptors that mediate cell migration via cell–cell and cell–matrix interactions^9^. They belong to a family of a cell surface adhesion molecules and are composed of α and β subunits that are non-covalently associated^10^. Integrin αvβ6 is the only heterodimer that the β6 subunit can form, and it is expressed exclusively in epithelial cells. It is absent or weakly expressed in healthy adult epithelia, but it is highly expressed during embryogenesis, tissue repair and carcinogenesis^11^,^12^. We have reported that β6 integrin played an important role in the invasiveness, metastasis and degradation of the extracellular matrix of colorectal cancer, thyroid carcinoma, gastric carcinoma, and pancreatic carcinoma, and it has been identified as an independent unfavorable prognostic indicator in a number of human cancers^11^,^13^,^14^,^15^,^16^,^17^,^18^,^19^. In addition, our previous research demonstrated that there is a direct link between extracellular signal-regulated kinase (ERK2) and β6 integrin, which is essential for β6-mediated ERK2 activation and the corresponding downstream effects^20^,^21^. Furthermore, we synthesized β6-targeted immunoliposomes and demonstrated that they had favorable antitumor efficacy and insignificant systemic toxicity in colon cancer^22^. Unsurprisingly, the exclusive expression of integrin β6 in epithelium-derived tumors and its important effects make it a novel therapeutic target for the treatment of many tumors. As cholangiocarcinoma originates from the ductular epithelium of the biliary tree, the expression and roles of integrin β6 in cholangiocarcinoma should be further explored.

Rac1 is a member of the Rho family of small GTPases. It is highly expressed in many cancer cell lines and has been implicated in a wide variety of cellular processes, including cytoskeletal reorganization and gene transcription, and its overexpression is correlated with poor prognosis in cholangiocarcinoma^23^,^24^. Furthermore, Rac1 also regulates various downstream effector molecules related to tumor invasiveness, such as MMP9 and uPA, and it is considered a central regulator of tumor malignancy^25^,^26^.

In the present study, for the first time, we demonstrated that integrin β6 is overexpressed in cholangiocarcinoma and is associated with lymph node metastasis. Our data also indicate that integrin β6 is crucial in the malignant progress of cholangiocarcinoma. Moreover, integrin β6 promotes the development of cholangiocarcinoma by activating Rac1. Our data indicated that integrin β6 may contribute to improving the diagnostic accuracy and treatment of cholangiocarcinoma and should be further explored.

## Results

### Integrin β6 expression was associated with lymph node metastasis and distant metastasis in cholangiocarcinoma

**Figure 1:**
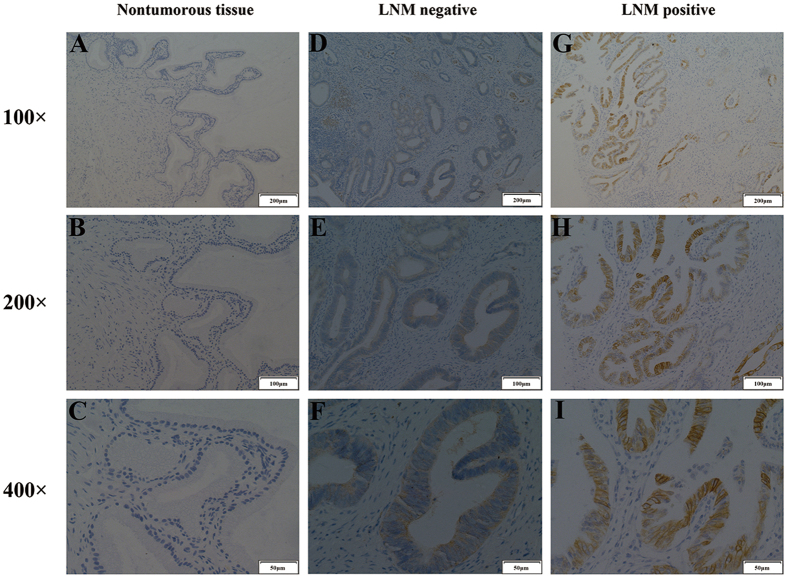
The expression of integrin β6 in non-tumorous bile duct tissues and cholangiocarcinoma tissues. (**A–C**) Negative β6 expression in non-tumorous bile duct tissues. (**D–F**) Moderate expression of integrin β6 in cholangiocarcinoma tissues without lymph node metastasis. (**G–I**) Strong staining of β6 in cholangiocarcinoma tissues with lymph node metastasis.

To investigate the expression of integrin β6 in cholangiocarcinoma, we performed an immunohistochemistry (IHC) analysis using a total of 95 paraffin-embedded cholangiocarcinoma samples and corresponding adjacent normal tissues. As shown in Fig. 1, integrin β6 staining was negative in non-tumorous tissues (Fig. 1A–C), while moderate expression was observed in tumor tissues (Fig. 1D–F). Furthermore, strong β6 expression was observed in specimens with lymph node metastasis (Fig. 1G–I). Integrin β6 was predominantly expressed in the membrane of the tumor cells. To further explore the clinical significance of β6 expression in cholangiocarcinoma, we analyzed the correlation between β6 expression and various clinicopathological factors. As shown in Table 1, no significant association was observed between β6 expression and age (*P* = 0.5226), gender (*P* = 0.6637), CA19-9 level (*P* = 0.2648), CEA level (*P* = 0.6670), depth of invasion (*P* = 0.4396) and tumor differentiation (*P* = 0.3684). However, β6 expression was significantly associated with lymph node metastasis (*P* = 0.0003) and distant metastasis (*P* = 0.0126), indicating that β6 may be involved in the progression of cholangiocarcinoma.

### Integrin β6 was a promising biomarker for diagnosis of cholangiocarcinoma and prediction of lymph node metastasis

**Figure 2:**
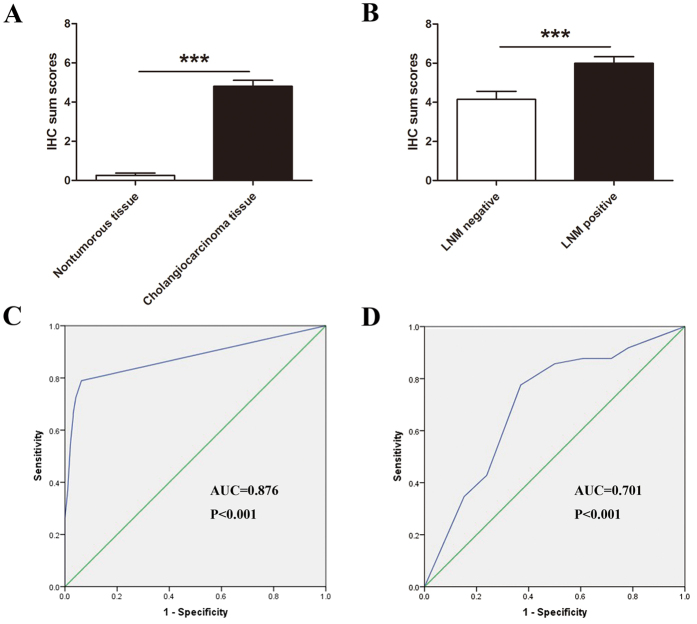
Upregulation of β6 in more aggressive cholangiocarcinoma tissues and ROC curves to assess the diagnostic value of β6 expression in cholangiocarcinoma. IHC sum scores were used to assess integrin β6 expression in cholangiocarcinoma tissues and adjacent non-tumorous tissues. β6 expression was significantly upregulated in cholangiocarcinoma tissues compared to adjacent non-tumorous tissues. (**A**) β6 expression was markedly higher in cholangiocarcinoma tissues with lymph node metastasis than those without lymph node metastasis. (**B**) The ROC curves showed strong separation between cholangiocarcinoma tissues and adjacent non-tumorous tissues, with an AUC of 0.989 (*P* < 0.001). (**C**) The ROC curves showed strong separation between the patients with and without lymph node metastasis, with an AUC of 0.741 (*P* < 0.001). ***P < 0.001.

As shown in Fig. 2A, β6 staining was negative in almost all non-tumorous tissues, while moderate to strong staining was observed in cholangiocarcinoma tissues, suggesting that β6 can be used for the diagnosis of cholangiocarcinoma. The IHC results showed that β6 expression was significantly upregulated in tumor tissues with lymph node metastasis compared to that of samples without lymph node metastasis (Fig. 2B).

To determine the diagnostic value of β6 expression in cholangiocarcinoma, we constructed receiver operator characteristic (ROC) curves. To assess the capacity of β6 expression (IHC sum scores) to diagnose cholangiocarcinoma or to predict lymph node metastasis, we calculated the area under the curve (AUC). The ROC curves suggested that the AUC value for the diagnosis of cholangiocarcinoma was up to 0.876 (Fig. 2C, CI (95%): 0.822 to 0.929, *P* < 0.001), with an estimated sensitivity and specificity of 78.95% and 93.68%, respectively, for β6 expression as a predictor of cholangiocarcinoma (Supplementary Table S1). Moreover, the AUC value for predicting lymph node metastasis in cholangiocarcinoma was 0.701 (Fig. 2D, CI (95%): 0.599 to 0.791, *P* < 0.001), with an estimated sensitivity and specificity of 77.55% and 63.04%, respectively (Supplementary Table S2).

### Integrin β6 markedly promoted the proliferation, migration and invasion of cholangiocarcinoma cells

**Figure 3:**
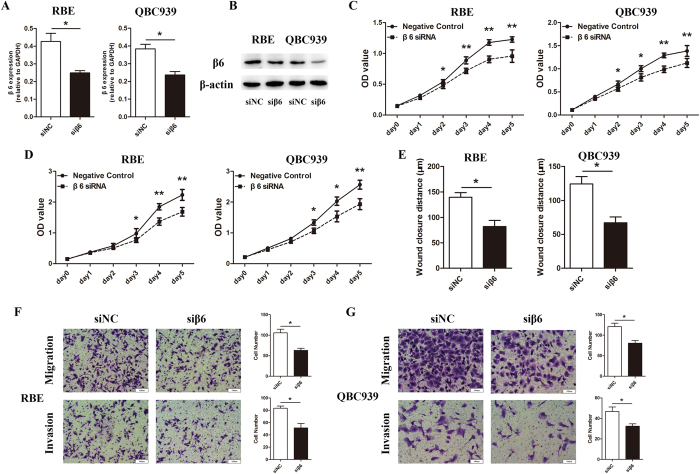
β6 silencing markedly decreased the proliferation, migration and invasion of cholangiocarcinoma cells. (**A**) Real-time PCR showed that integrin β6 expression was high in both RBE and QBC939 cells; β6 expression was significantly suppressed after β6-specific siRNA transfection. (**B**) Western blotting showed that β6 expression was significantly suppressed after β6 siRNA transfection in RBE and QBC939 cells at the protein level. (**C**) CCK8 assays revealed that silencing of β6 significantly decreased cell viability in RBE and QBC939 cells. (**D**) After transfection of β6 siRNA, MTT assays were conducted to evaluate the proliferation of RBE and QBC939 cells. (**E**) RBE and QBC939 cells transfected with β6 siRNA were used for wound-healing assays. (**F,G**) RBE (**F**) and QBC939 (**G**) cells transfected with β6 siRNA were used for Transwell migration assays and invasion assays. Five fields of cells in the lower chamber were counted (×200 magnification). Data represent the mean ± SD of three independent experiments. *P < 0.05; **P < 0.01.

As integrin β6 was highly expressed in cholangiocarcinoma, the functional roles of integrin β6 in human cholangiocarcinoma cells were investigated. β6-specific siRNA was used in RBE and QBC939 cells. First, the efficiency of β6 silencing in these cells was determined. As shown in Fig. 3A,B, β6 expression was significantly decreased at both the mRNA level and protein level after transfection with β6-specific siRNA. CCK8 assays were used to evaluate the effects of integrin β6 on the viability of cholangiocarcinoma cells. The results showed that β6 silencing in RBE and QBC939 cells markedly suppressed cell viability 48 hours after transfection in a time-dependent manner (Fig. 3C). Then, MTT assays were conducted, and the results indicated that β6 downregulation in RBE and QBC939 cells was associated with decreased cell proliferation (Fig. 3D). Wound-healing assays showed that the migration of RBE and QBC939 cells was markedly suppressed after downregulation of β6 (Fig. 3E). Then, we performed Transwell migration and invasion assays in cholangiocarcinoma cells after β6 silencing. β6 silencing had no obvious effect on cell viability within 12 hours, which eliminated the potential confounding influence of β6 silencing-induced cell proliferation on cell migration and invasion. The results showed that the migration and invasion capacities of both RBE and QBC939 cells were significantly suppressed by β6 silencing (Fig. 3F,G).

**Figure 4:**
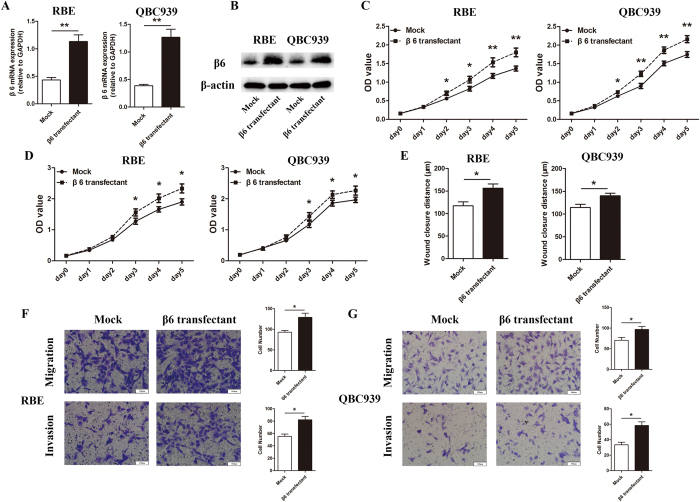
β6 overexpression markedly promoted the proliferation, migration and invasion of cholangiocarcinoma cells. (**A**) Real-time PCR showed that integrin β6 expression was significantly upregulated after transfection of the β6 overexpression plasmid. (**B**) Western blotting showed that β6 expression was significantly increased after transfection with the β6 overexpression plasmid in RBE and QBC939 cells at the protein level. (**C**) CCK8 assays revealed that overexpression of β6 significantly promoted cell viability in RBE and QBC939 cells. (**D**) After β6 overexpression, MTT assays were conducted to evaluate the proliferation of RBE and QBC939 cells. (**E**) RBE and QBC939 cells transfected with the β6 overexpression plasmid were used for wound-healing assays. (**F,G**) RBE (**F**) and QBC939 (**G**) cells transfected with the β6 overexpression plasmid were used for Transwell migration assays and invasion assays. Five fields of cells were counted in the lower chambers (×200 magnification). Data represent the mean ± SD of three independent experiments. *P < 0.05; **P < 0.01.

To further confirm the effects of β6 on cell proliferation, migration and invasion in cholangiocarcinoma cells, we repeated the aforementioned experiments after upregulation of β6 using a β6 overexpression plasmid. As shown in Fig. 4A,B, β6 expression was significantly increased after transfection. The results showed that β6 overexpression markedly promoted the proliferation, migration and invasion of cholangiocarcinoma cells (Fig. 4C–G).

These data suggested that integrin β6 could promote the proliferation, migration and invasion of cholangiocarcinoma cells.

### Integrin β6 induced the expression of MMP9 in cholangiocarcinoma cells

**Figure 5:**
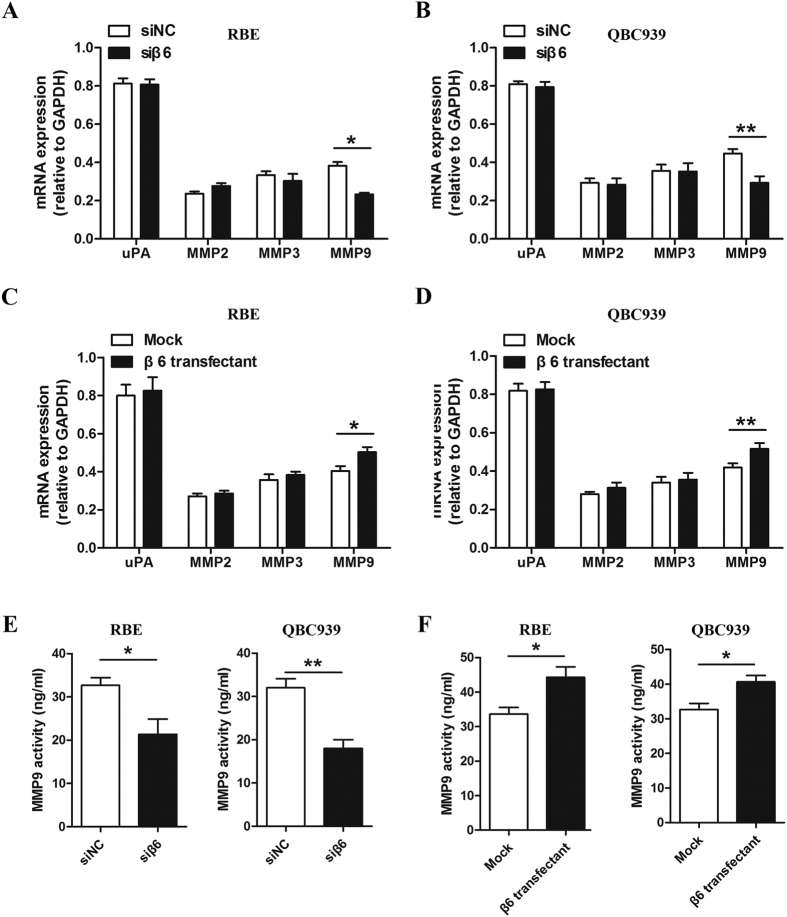
Integrin β6 increased MMP9 expression at both the mRNA and protein levels. (**A,B**) MMP9 mRNA was significantly decreased in RBE cells (**A**) and QBC939 cells (**B**) after transfection with β6 siRNA, while the expression of uPA, MMP2 and MMP3 showed no significant changes. (**C,D**) MMP9 mRNA was significantly increased in RBE cells (**C**) and QBC939 cells (**D**) after β6 overexpression, while the expression of uPA, MMP2 and MMP3 showed no significant changes. (**E**) The protein level of MMP9 was detected by ELISA in RBE and QBC939 cells transfected with β6 siRNA. (**F**) The protein level of MMP9 was detected by ELISA in RBE and QBC939 cells after β6 overexpression. Data represent the mean ± SD from three independent experiments. *P < 0.05; **P < 0.01.

To identify the mechanism underlying the effects of β6 on invasion in cholangiocarcinoma cells, we measured the mRNA expression of uPA, MMP2, MMP3, and MMP9 using quantitative real-time PCR after silencing or overexpressing β6. We found that β6-specific siRNA significantly downregulated MMP9 mRNA expression in cholangiocarcinoma cells, and β6 overexpression markedly increased MMP9 mRNA expression. No effect was observed on uPA, MMP2 and MMP3 (Fig. 5A–D).

We further explored the effect of β6 on secreted MMP9 in the supernatant of cholangiocarcinoma cells by ELISA. Consistent with the results from the mRNA analysis, the data showed that the secreted MMP9 in the supernatant of cholangiocarcinoma cells was significantly decreased after suppression of β6 expression (Fig. 5E), while β6 overexpression increased MMP9 secretion in the supernatant (Fig. 5F). Taken together, these results revealed that integrin β6 could induce MMP9 expression at both the mRNA and protein levels.

### Integrin β6 activated Rac1 in cholangiocarcinoma cells

**Figure 6:**
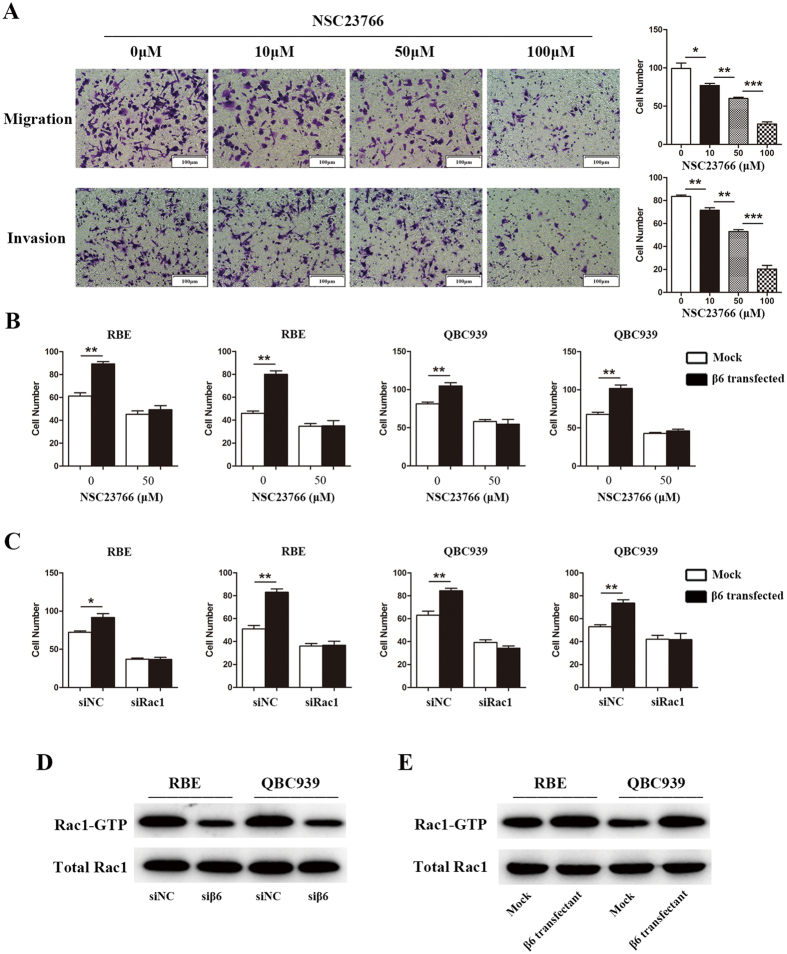
Integrin β6 promoted the invasiveness of cholangiocarcinoma cells by activating Rac1. (**A**) RBE cells treated with different concentrations of NSC23766, a Rac1 inhibitor, were used for migration and invasion assays. (**B**) After treatment with NSC23766, RBE and QBC939 cells were transfected with the β6-overexpressing plasmid, and the migration and invasion capacities were measured by Transwell assays. (**C**) RBE and QBC939 cells co-transfected with Rac1-specific siRNA and β6-overexpressing plasmid were used for migration and invasion assays. (**D**) After transfection with β6 siRNA or β6-overexpressing plasmid, cell lysates of RBE and QBC939 were used for PAK-GST pull down assays. Activated Rac1 as well as total Rac1 pulled down in the lysates were detected by western blot analysis. Data are shown as the mean ± SD, and the results shown are representative of 3 independent experiments. Five fields of cells in the lower compartment were counted in Transwell assays (200× magnification). *P < 0.05; **P < 0.01; ***P < 0.001.

As previous studies showed that Rac1 activity is involved in the malignant behaviors of cancer cells^24^, we hypothesized that integrin β6 may promote invasiveness by activating Rac1. To confirm our hypothesis, we performed four experiments. First, using the specific Rac1 activity inhibitor NSC23766, we found that Rac1 was essential for the migration and invasion of cholangiocarcinoma cells, as NSC23766 suppressed migration and invasion of RBE cells in a dose-dependent manner (Fig. 6A). Then, we demonstrated that overexpression of integrin β6 could effectively promote the migration and invasion capacity of cholangiocarcinoma cells, whereas NSC23766 significantly inhibited this effect (Fig. 6B). Moreover, the β6-mediated promotion of migration and invasion could be inhibited by specific Rac1 siRNA (Fig. 6C). In addition, we found that β6 silencing significantly decreased Rac1-GTPase activity (Fig. 6D), while β6 overexpression increased Rac1 activity in cholangiocarcinoma cells (Fig. 6E). All these results demonstrated that β6 promoted migration and invasion by activating Rac1.

### The effects of integrin β6 were mediated through Rac1 downstream effectors, F-actin polymerization and MMP9 expression in cholangiocarcinoma cells

**Figure 7:**
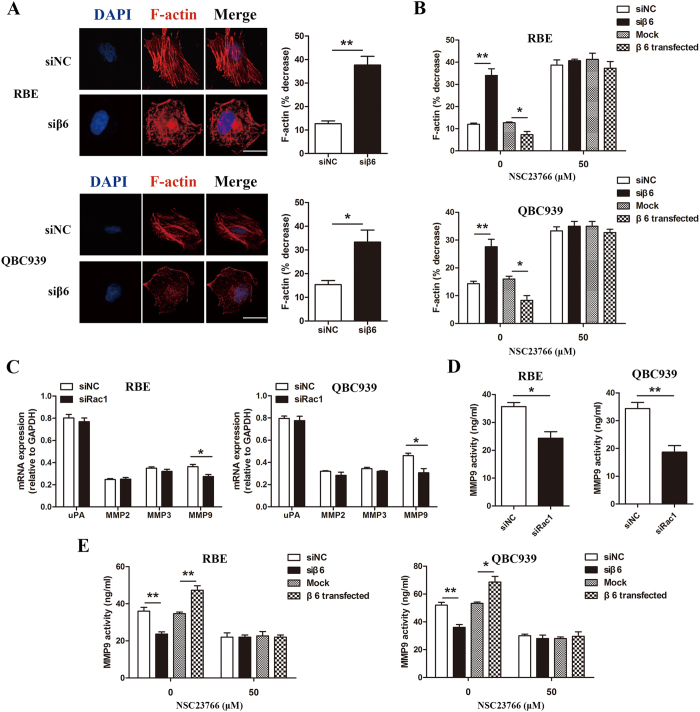
Integrin β6 induced F-actin polymerization and MMP9 expression by activating Rac1. (**A**) After transfection of β6 siRNA, F-actin in RBE and QBC939 cells was stained by TRITC-conjugated phalloidin and observed with laser confocal microscopy. The % decrease in F-actin = [(F-actin in untreated cells–F-actin in NC or β6 siRNA treated cells)/F-actin in untreated cells] ×100. Scale bar, 10 μm. (**B**) After treatment with NSC23766, RBE and QBC939 cells were transfected with β6 siRNA or β6-overexpressing plasmid, and F-actin was stained by TRITC-conjugated phalloidin and observed with laser confocal microscopy. (**C**) MMP9 mRNA expression was significantly decreased in RBE cells and QBC939 cells after transfection with Rac1 siRNA, while the expression of uPA, MMP2 and MMP3 showed no significant changes. (**D**) The protein level of MMP9 was detected by ELISA in RBE and QBC939 cells after Rac1 silencing. (**E**) The protein level of MMP9 was detected by ELISA in RBE (**C**) and QBC939 cells (**D**) pretreated with NSC23766 and β6 siRNA or β6-overexpressing plasmid. Data represent the mean ± SD from three independent experiments. *P < 0.05; **P < 0.01.

Previous studies found that Rac1 activity is important for microfilament dynamics, which directly regulate cell motility^27^. In the present study, we explored the effect of integrin β6 on the microfilament dynamics in cholangiocarcinoma cells. Figure 7A shows that in the siNC group, F-actin was organized into the cytoskeleton, while in the β6-silenced group, F-actin was depolymerized in most cells. The results revealed that F-actin was significantly depolymerized after treatment with β6 siRNA. Moreover, β6 overexpression increased F-actin polymerization, and after treatment with NSC23766, this effect was abolished (Fig. 7B), indicating that β6 promoted F-actin polymerization by activating Rac1.

Our previous work showed that β6 induced the expression of MMP9 and promoted the invasiveness of cholangiocarcinoma cells in a Rac1-dependent manner. We thus hypothesized that integrin β6 induced the expression of MMP9 via the Rac1 pathway. To test our hypothesis, we explored the effect of Rac1 on β6-induced MMP9 expression. Rac1 silencing significantly decreased the expression of MMP9 at both the mRNA and protein level (Fig. 7C,D). Moreover, β6 silencing decreased MMP9 expression, while β6 overexpression increased the expression of MMP9. However, after pretreatment with a Rac1 activity inhibitor, the effect of both β6 silencing and overexpression was abolished (Fig. 7E). These results confirmed our hypothesis and indicated that integrin β6 induced the expression of MMP9 by activating Rac1.

### Integrin β6 promoted tumor growth and MMP9 expression of tumor cells in nude mice

**Figure 8:**
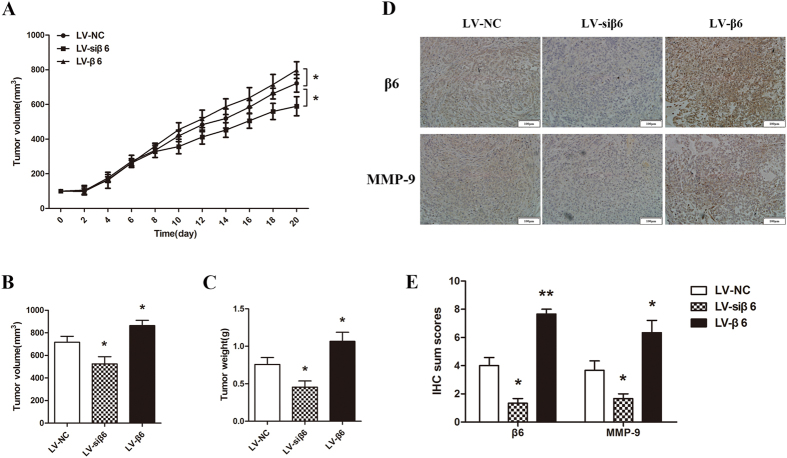
Integrin β6 effectively promoted tumor growth and MMP9 expression of subcutaneous xenograft tumors. The growth curves of tumors from the LV-NC, LV-siβ6 and LV-β6 groups. Tumors were isolated and measured. The mean volume of tumors in the LV-siβ6 group (n = 6) was significantly smaller than that of the control, while the LV-β6 group (n = 6) had a larger mean tumor volume. (**A**) The mean tumor weight was significantly lower in the LV-siβ6 group (n = 6) and higher in the LV-β6 group (n = 6) compared to the control. (**B**) The expression of integrin β6 and MMP9 was detected by IHC in paraffin-embedded tumor tissue sections. (**C**) IHC sum scores were used to determine the expression of integrin β6 and MMP9 in the xenograft tumor tissues. Data represent the mean ± SD. *P < 0.05; **P < 0.01.

To evaluate the effect of integrin β6 *in vivo*, we established a subcutaneous xenograft tumor model in nude mice bearing RBE cells. RBE cells were infected with the lentiviral vectors LV-negative control, LV-siβ6 or LV-β6. Consistent with our *in vitro* findings, our results showed that silencing of β6 markedly suppressed the growth of RBE xenograft tumors compared with that of the NC group, while β6 overexpression promoted the growth of tumors (Fig. 8A). The overall mean tumor volume and tumor weight of the LV-siβ6 group were significantly smaller than those of the LV-NC group, whereas tumors in the LV-β6 group had a relatively larger volume and weight (Fig. 8B,C). As shown in Fig. 8D,E, compared with the LV-NC group, β6 silencing markedly decreased the MMP9 expression in the xenograft tumors, while β6 overexpression significantly increased the MMP9 expression. These results indicated that integrin β6 promoted the growth and MMP9 expression of cholangiocarcinoma xenograft tumors.

## Discussion

Cholangiocarcinoma is a predominantly fatal cancer, which is usually difficult to diagnose and to treat^7^. Therefore, elucidating the mechanisms underlying the malignant behavior of cholangiocarcinoma may facilitate the development of clinical strategies. In the present study, β6 expression was markedly upregulated in cholangiocarcinoma and was associated with lymph node metastasis. Integrin β6 promoted the migratory and invasive capacities of cholangiocarcinoma cells by activating Rac1, indicating that integrin β6 may be a potential target for the treatment of cholangiocarcinoma.

The epithelial-restricted integrin β6 is generally upregulated in tissues that have undergone malignant transformation^28^,^29^. Increased expressions of β6 were previously reported in carcinomas of the colon, breast, stomach and pancreas, and increased β6 expression is associated with advanced tumor stage and poor prognosis^13^,^17^,^18^,^30^. Patsenker *et al*. reported that integrin β6 is a highly specific immunohistochemical marker for the differential diagnosis of hepatocellular carcinoma (HCC) and cholangiocarcinoma, as β6 is strongly expressed in cholangiocarcinoma, and in HCC, β6 staining is negative^31^. Here, we reported that the increased β6 expression was related to lymph node metastasis and distant metastasis, suggesting that in addition to an immunohistochemical marker, β6 staining in the surgical specimens could be used to evaluate the risks of lymph node metastasis. Additionally, integrin β6 may also be involved in the development of cholangiocarcinoma.

Integrin β6 promotes malignant behaviors, such as cell proliferation, migration, invasion and metastasis, in many types of malignancies^15^,^17^,^21^,^30^. However, the role of integrin β6 in cholangiocarcinoma cells has not been elucidated. Our results demonstrated that β6 could significantly promote cell proliferation and invasion.

Previous studies showed that β6 increases the expression and secretion of MMP2, MMP3 and MMP9, resulting in the degradation of the extracellular matrix and increased tumor cell invasiveness in colon cancer, pancreatic cancer and ovarian cancer^32^,^33^. MMP9 is highly expressed in tissues, and it was shown to be a valuable prognostic indicator in cholangiocarcinoma^34^. As β6 also promoted the invasiveness of cholangiocarcinoma cells, we explored the effect of β6 on MMP expression in cholangiocarcinoma. Additionally, the effect of β6 on uPA was assessed due to its role in cell invasiveness, and uPA was highly expressed in cholangiocarcinoma cells. Our results showed that β6 induced the expression of MMP9 but not that of uPA, MMP2 or MMP3 in cholangiocarcinoma cells. The detailed mechanism by which β6 promotes tumor development is complicated. Integrin β6 may functions through the protein kinase C (PKC) pathway in colon cancer, while β6 may also bind ERK and subsequently activate the ERK-Ets1 pathway in colon cancer and pancreatic cancer^35^. The SDF-1/CXCR4 axis and IL-8 are also involved in the β6-regulated tumor cell migration^15^,^36^. Rac1 plays a vital role in the development of cholangiocarcinoma^24^,^37^. Rac1 is a member of the Rho family of small GTPases and has been implicated in a wide variety of cellular processes, especially cell motility and cytoskeleton polymerization. Rac1 could also induce the expression and secretion of MMPs^23^,^25^,^38^,^39^. Therefore, targeting Rac1 may be a promising therapeutic approach for the treatment of cholangiocarcinoma. MMP9 was reported to contribute to the cholangiocarcinogenesis in a Rac1-dependent manner^40^. Consistent with these results, our data demonstrated that β6 promoted the invasiveness of cholangiocarcinoma cells by activating Rac1, as both NSC23766 and Rac1 siRNA could abolish the effect of β6. In addition, β6 could activate Rac1, but it had no effect on total Rac1 expression. Mechanistically, we demonstrated that β6 facilitated F-actin polymerization and induced MMP9 expression by activating Rac1. These results indicated that integrin β6 is an endogenous activator of Rac1 in cholangiocarcinoma, which contributed to the malignant behaviors of cholangiocarcinoma cells.

Previous studies reported that integrin β6 could localize to the cytoplasm and activate ERK via a specific binding site. Then, activated ERK translocates to the nucleus and activates Ets-1, leading to the proliferation, migration, and invasion of tumor cells^20^,^32^,^35^. Here, we demonstrated that β6 promoted tumor cell malignant behaviors by activating Rac1. Rac1 is important in regulating vesicular transport. Therefore, the effect of Rac1 on the internalization of β6 needs to be further explored.

Cholangiocarcinoma is a severe disease with poor prognosis. Molecular targeting strategies to improve treatment and reduce the side effects of conventional chemotherapy and radiotherapy are needed. In the present study, we demonstrated that integrin β6 upregulation was an unfavorable indicator in cholangiocarcinoma. By targeting and activating Rac1, β6 promotes the development and progression of cholangiocarcinoma. The perturbation of integrin β6 may present a potential strategy for the treatment of patients suffering from cholangiocarcinoma.

## Materials and Methods

### Tissue samples

A total of 95 human cholangiocarcinoma specimens and the corresponding adjacent normal specimens were collected from Qilu Hospital of Shandong University. Among the 95 cholangiocarcinoma specimens, 61 cases had lymph node metastasis, while 34 cases had no lymph node metastasis. Clinicopathological classification and staging were determined according to the American Joint Committee on Cancer (AJCC) criteria^41^. All samples were fixed in 40 g/L formaldehyde and were embedded in paraffin for histological diagnosis and immunohistochemical studies. The protocols in the study were carried out in accordance with the approved guidelines. All the experimental protocols were approved by the Ethics Committee of Shandong University, China. Written informed consent was obtained from all subjects.

### Cell culture and transfection

The human cholangiocarcinoma cell lines, RBE and QBC939, obtained from the American Type Culture Collection (ATCC), were separately maintained in RPMI 1640 and DMEM medium, respectively, (Gibco, CA, USA) supplemented with 10% inactivated fetal bovine serum (FBS) (Gibco, CA, USA) in a humidified cell incubator with an atmosphere of 5% CO_2_ at 37 °C. Both RBE and QBC939 cells were transfected with β6 siRNA or a β6 overexpression plasmid using Lipofectamine 2000 according to the manufacturer’s protocols (Invitrogen, Carlsbad, CA, USA). The β6 overexpression plasmid was constructed as previously described^20^. The β6 siRNA primers were purchased from GenePharma and were as follows: 5′-GCUAAAGGAUGUCAAUUAATT-3′, reverse 5′-UUAAUUGACAUCCUUUAGCTA-3′. The Rac1-siRNA were as follows: 5′-GCAAACAGAUGUGUUCUUA-3′, reverse 5′-UAAGAACACAUCUGUUUGC-3′. Nonspecific negative control primers (forward 5′-UUCUCCGAACGUGUCACGUTT-3′, reverse 5′-ACGUGACACGUUCGGAGAATT-3′) were purchased from Sigma-Aldrich (St. Louis, USA).

### RNA isolation and real-time quantitative PCR

Total RNAs were extracted from transfected cells using TRIzol reagent (Invitrogen, Carlsbad, CA, USA) and were reverse-transcribed into cDNA using a ReverTra Ace qPCR Kit (Toyobo, Osaka, Japan). Real-time PCR was performed using an UltraSYBR Mixture (CWBIO). The sequences of the sense and antisense primers were as follows: β6 integrin forward: 5′-AGGATAGTTCTGTTTCCTGC-3′, and reverse: 5′-ATCATAGGAATATTTGGAGG-3′; GAPDH: 5′-AACGGATTTGGTCGTATTGGG-3′, and reverse 5′-CCTGGAAGATGGTGATGGGAT-3′; MMP-2 forward: 5′-TGATCTTGACCAGAATACCATCGA-3′, and reverse: 5′-GGCTTGCGAGGGAAGAAGTT-3′; MMP-3 forward: 5′-CGGTTCCGCCTGTCTCAAG-3′ and reverse: 5′-CGCCAAAAGTGCCTGTCTT-3′; MMP-9 forward: 5′-ACCTCGAACTTTGACAGCGAC-3′, and reverse: 5′-GAGGAATGATCTAAGCCCAGC-3′. Relative gene expression levels were normalized to GAPDH as a control.

### Western blotting

After 48 hours of transfection, cells were subjected to protein extraction using cell lysis buffer containing 1% protease inhibitors. Protein concentrations of the homogenized lysates were measured using a BCA protein assay kit (Sangon, Shanghai, China). Aliquots containing 30 μg of protein were separated by 10% SDS-PAGE and then transferred to PVDF membranes (Millipore, Billerica, MA, USA). Membranes were incubated overnight at 4 °C with the following primary antibodies: mouse monoclonal antibody against integrin β6 (1:1000; Millipore, CA, USA), mouse monoclonal antibody against Rac1 (1:300; Abcam, Hong Kong), or anti-β-actin (1:1000; ZSGB-Bio, Beijing, China), followed by secondary antibodies (1:2000; goat-anti rabbit or mouse IgG, ZSGB-Bio) conjugated to peroxidase for 1 hour at room temperature. After washing, immunoreactivity was visualized using an enhanced chemiluminescence kit (Millipore, Billerica, MA, USA) according to the manufacturer’s instructions.

### Immunohistochemistry (IHC)

IHC was performed using paraffin-embedded tissue sections. The sections were dewaxed and hydrated, followed by antigen retrieval (in 0.01 mol/L citrate buffer solution, pH 6.0, heated to boiling for 2–3 min in a stainless steel pressure cooker). Endogenous peroxidase was blocked using a 3% hydrogen peroxide solution. The section was incubated with the blocking goat serum for 15 min and then immunostained with rabbit antibody against β6 (dilution 1:500) at 4 °C overnight. Secondary staining was performed with HRP-conjugated anti-rabbit IgG using a MaxVision Kit and a 3, 5-diaminobenzidine (DAB) peroxidase substrate kit (Maixin Co, Fuzhou, China). The sections were then counterstained with hematoxylin.

### Evaluation of immunohistochemical staining

Immunohistochemical staining was independently evaluated by two experienced pathologists in a blinded manner. Staining was semi-quantitatively scored based on both the staining intensity (0, negative; 1, weak; 2, moderate; 3, strong) and the percentage of positively stained cells (0, 0%; 1, 1–25%; 2, 26–50%; 3, 51–75%; 4, 76–100%). Both scores for each specimen were then combined to obtain the final score of β6 expression. The cut-off point for the sum of the scores was defined as follows: 0–2, low expression; 3–7, high expression. The appropriateness of the cut-off point was validated by ROC analysis.

### Cell viability assays

A total number of 2500 cells was seeded in 96-well plates in triplicate wells and cultured for the indicated times. Cell viability was evaluated using the Cell Counting Kit-8 (CCK8) (Beyotime, Haimen, China) assay according to the manufacturer’s instructions. The absorbance was determined at 450 nm. Each time point was replicated in three wells, and the experiment was independently performed at least three times.

### Cell proliferation assays

Cell proliferation assays were performed using MTT (3-(4,5-dimethylthiazol-2-yl)-2,5-diphenyl tetrazolium bromide). A total number of 2500 cells was seeded in 96-well plates. Then, 24 hours after β6 silencing or overexpression, MTT at 5 mg/ml was added and incubated for 4 hours. Finally, the medium was aspirated, DMSO was added, and the absorbance was determined at 490 nm. Each time point was repeated in three wells, and the experiment was independently performed at least three times.

### Wound-healing assays

The monolayer wound-healing assay was used to assess cell migration. A total of 5 × 10^5^ cells were seeded in 6-well plates, incubated overnight, and then pretreated with β6 siRNA or the β6 overexpression plasmid. After achieving 90% confluency, the cell monolayer was scratched with a sterile pipette tip, the floating cells were removed with PBS, and the cells were cultured for 24 hours. Photographic images were taken at 0 and 24 hours along the scrape line using a microscope. The results are expressed as the actual wound closure distance.

### Transwell assays for cell migration and invasion

Tumor cell migration assays were analyzed in 24-well Boyden chambers with 8-μm pore size polycarbonate membranes (Costar, Acton, USA). For invasion assays, the membranes were precoated with 50 μg Matrigel (BD Biosciences, San Diego, USA) to simulate matrix barriers. Then, 1 × 10^5^ cells were resuspended in 200 μl serum-free medium and seeded into the upper chamber, and the lower compartments were filled with 600 μl medium with 10% FBS. After incubation (incubation time for migration is 10–12 hours and for invasion is 24 hours), the upper surface of the Transwell membrane was wiped gently with a cotton swab to remove the non-migrating cells. The membranes were fixed with methanol and stained with 0.1% crystal violet for twenty minutes. The stained cells were counted under a light microscope at × 200 magnification in at least five fields. NSC23766 (Calbiochem, San Diego, USA), a specific Rac1 inhibitor, was used to inhibit Rac1 activity in several experiments.

### MMP activity assays

A total of 2 × 10^5^ cells were cultured in a six-well culture plate and then treated with siNC, siβ6, mock plasmid or β6 overexpression plasmid for 24 hours. The levels of secreted MMP-9 in the culture supernatant were determined using an enzyme-linked immunosorbent assay (ELISA) following the manufacturer’s ELISA kit guidelines (R&D). Samples were assayed in triplicate and calibrated against a standard curve.

### Immunofluorescence for F-actin staining

To observe the effect of integrin β6 on F-actin, cholangiocarcinoma cells were seeded (1 × 10^4^) on the cover slip and cultured overnight. Twenty-four hours after transfection, cells were fixed, permeabilized and then stained with tetramethylrhodamine (TRITC)-conjugated phalloidin (Sigma–Aldrich, St. Louis, USA) for 1 hour. Nuclei were stained with 4′, 6-diamidino-2-phenylindole (DAPI) (Beyotime) for 5 min. The results were analyzed with confocal laser microscopy (Carl Zeiss, LSM780, Oberkochen, Germany) and are expressed as the % decrease in F-actin = [(F-actin in untreated cells–F-actin in siNC or siβ6 treated cells)/F-actin in untreated cells] ×100.

### Rac1 activity assay

The levels of the active GTP-bound form of Rac1 were determined with p21-activated kinase (PAK)-GST protein beads (Cytoskeleton). Cell lysate was prepared as previously described^42^. The lysate was incubated with 20 μg of PAK-GST protein beads for 30 min at 4 °C. Proteins on beads and in total cell lysates were assessed by SDS-PAGE followed by immunoblotting with a Rac1-antibody.

### Xenograft tumor model

BALB/c nude mice (4–6 weeks old) were obtained from the Chinese Academy of Sciences (Shanghai, China) and maintained in laminar-flow cabinets under specific pathogen-free conditions. Cultured RBE cells were infected with a lentiviral vector LV-negative control, LV-siβ6 or LV-β6 (Genechem, Shanghai) using a standard protocol. To evaluate tumor growth *in vivo*, 1 × 10^7^ RBE cells were injected subcutaneously into the flanks of nude mice. The tumor size was measured every three days, and the tumor volume was calculated as follows: longer diameter × shorter diameter^2^ × 0.5. All mice were sacrificed after 22 days. Subcutaneous tumor grafts were excised and weighed. Then, the tumors were processed for standard IHC analysis. All animal studies were conducted using a protocol approved by the Institutional Animal Care and Use Committee of the School of Medicine, Shandong University (Jinan, Shandong, China).

### Statistical analysis

The associations between β6 expression and clinicopathological parameters were analyzed by the chi-square test and Fisher’s exact test. Quantitative data are presented as the mean ± SD. The statistical significance was determined by two-tailed paired Student’s t-test in two groups and one-way ANOVA in multiple groups. Receiver operating characteristic (ROC) curve analysis was performed to assess the diagnostic value of β6 in cholangiocarcinoma. All statistical analyses were performed using SPSS 18.0 software (SPSS Inc., Chicago, USA), and a *P* value < 0.05 was considered statistically significant.

## Additional Information

**How to cite this article**: Li, Z. *et al*. Integrin β6 serves as an immunohistochemical marker for lymph node metastasis and promotes cell invasiveness in cholangiocarcinoma. *Sci. Rep.* **6**, 30081; doi: 10.1038/srep30081 (2016).

## Supplementary Material

Supplementary Information

## Figures and Tables

**Table 1:** Correlations between β6 expression and clinicopathological characteristics in cholangiocarcinoma.

Variable	Number	β6 expression		P value
		Low	High	
**Age (years)**				
<60	42	13	29	0.5226
≥60	53	20	33	
**Gender**				
Male	57	21	36	0.6637
Female	38	12	26	
**CA19-9 level**				
<37 ku/ml	35	15	20	0.2648
≥37 ku/ml	60	18	42	
**CEA level**				
<15 ng/ml	40	15	25	0.6670
>15 ng/ml	55	18	37	0.4396
**Depth of invasion (T)**				
Tis	3	2	1	
T1	16	7	9	
T2	38	13	25	
T3	36	10	26	
Missing	2	1	1	
**Lymph node metastasis (LNM)**				**0.0003**
Negative	34	20	14	
Positive	61	13	48	
**Distant metastasis (M)**				
Negative (M0)	72	30	42	**0.0126**
Positive (M1)	23	3	20	
**Tumor differentiation**				
Well	15	3	12	0.3684
Moderate	37	12	25	
Poor	40	16	24	
Missing	3	2	1	
