# Supplementary material for: Integrin β6 serves as an immunohistochemical marker for lymph node metastasis and promotes cell invasiveness in cholangiocarcinoma
Source: Sci Rep. 2016 Jul 21;6:30081. doi: 10.1038/srep30081 (PMC4954992; doi:10.1038/srep30081)
Supplement: Supplementary Information [file srep30081-s1.doc]

**Supplemental Data**

**Title:** Integrin β6 serves as an immunohistochemical marker for lymph node metastasis and promotes cell invasiveness in cholangiocarcinoma

**Author:** Zequn Li^1,2^, Siddhartha Biswas^1,2^, Benjia Liang^1,2^, Xueqing Zou^1,2^, Liqun Shan^1,2^, Yang Li^1,2^, Ruliang Fang^1^, Jun Niu^1^*

| **Supplementary Table S1: Sensitivity, Specificity, and Positive and Negative Predictive Values for cholangiocarcinoma detection using IHC scores of integrin β6 expression** | | | | | | | | |
| --- | --- | --- | --- | --- | --- | --- | --- | --- |
| **Criterion** | **Sensitivity** | **95%CI** | **Specificity** | **95%CI** | **PPV^a^** | **95%CI** | **NPV^b^** | **95%CI** |
| **>0** | **78.95** | **69.4-86.6** | **93.68** | **86.8-97.6** | **92.6** | **84.5-97.3** | **81.7** | **73.1-88.4** |

**^a^PPV= Positive Predictive Value**

**^b^NPV= Negative Predictive Value**

| **Supplementary Table S2: Sensitivity, Specificity, and Positive and Negative Predictive Values for LNM^a^ detection using IHC scores of integrin β6 expression** | | | | | | | | | |
| --- | --- | --- | --- | --- | --- | --- | --- | --- | --- |
| **Criterion** | **Sensitivity** | **95%CI** | **Specificity** | **95%CI** | **PPV^b^** | **95%CI** | **NPV^c^** | **95%CI** |  |
| **>5** | **77.55** | **63.4-88.3** | **63.04** | **47.5-76.8** | **69.1** | **55.0-81.0** | **72.5** | **56.1-85.4** |  |

**^a^LNM= Lymph node metastasis**

**^b^PPV= Positive Predictive Value**

**^c^NPV= Negative Predictive Value**
